# Comparative Transcriptome Analysis Reveal Candidate Genes Potentially Involved in Regulation of Primocane Apex Rooting in Raspberry (*Rubus* spp.)

**DOI:** 10.3389/fpls.2017.01036

**Published:** 2017-06-13

**Authors:** Jianfeng Liu, Yuetong Ming, Yunqing Cheng, Yuchu Zhang, Jiyang Xing, Yuqi Sun

**Affiliations:** Jilin Provincial Key Laboratory of Plant Resource Science and Green Production, Jilin Normal UniversitySiping, China

**Keywords:** *Rubus*, adventitious roots, transcriptome

## Abstract

Raspberries (*Rubus* spp.) exhibit a unique rooting process that is initiated from the stem apex of primocane, conferring an unusual asexual mode of reproduction to this plant. However, the full complement of genes involved in this process has not been identified. To this end, the present study analyzed the transcriptomes of the *Rubus* primocane and floricane stem apex at three developmental stages by Digital Gene Expression profiling to identify genes that regulate rooting. Sequencing and de novo assembly yielded 26.82 Gb of nucleotides and 59,173 unigenes; 498, 7,346, 4,110, 7,900, 9,397, and 4,776 differently expressed genes were identified in paired comparisons of SAF1 (floricane at developmental stage 1) vs. SAP1 (primocane at developmental stage 1), SAF2 vs. SAP2, SAF3 vs. SAP3, SAP1 vs. SAP2, SAP1 vs. SAP3, and SAP2 vs. SAP3, respectively. SAP1 maintains an extension growth pattern; SAP2 then exhibits growth arrest and vertical (downward) gravitropic deflection; and finally, short roots begin to form on the apex of SAP3. The Kyoto Encyclopedia of Genes and Genomes enrichment analysis of SAP1 vs. SAP2 revealed 12 pathways that were activated in response to shoot growth arrest and root differentiation, including circadian rhythm—plant (ko04712) and plant hormone signal transduction (ko04075). Our results indicate that genes related to circadian rhythm, ethylene and auxin signaling, shoot growth, and root development are potentially involved in the regulation of primocane apex rooting in *Rubus*. These findings provide a basis for elucidating the molecular mechanisms of primocane apex rooting in this economically valuable crop.

## Introduction

Raspberries (*Rubus* sp.) are an important economic fruit crop that grows in all temperate regions of the world. The raspberry fruit contains abundant polyphenol antioxidants, including anthocyanin pigments that are important ingredients of health products and can potentially prevent various human diseases (Skrovankova et al., 2015). Raspberries have a unique rooting characteristic, and its biennial shoots can grow several meters and root at the stem apex in autumn (Heslop-Harrison, 1959). In order to prevent excessive vegetative spread via rooting at stem apices, newly planted red raspberries often require staking to hold them upright. The apex rooting process of the stem apex can be divided into three successive stages: in stage 1, the stem exhibits an elongation growth pattern; in stage 2, elongation growth ceases and is followed by gravitropic curvature of the stem; and in stage 3, the root primordium differentiates from the stem apex and the root extends. After the formation and growth of new root at the stem apex, negatively geotropic shoots form at the rooting boss. New plants are generated in the next growth season. Thus, not only root primordium differentiation but also growth arrest and geotropism deflection are involved in formation of adventitious roots (ARs) at the stem apex, in contrast to adventitious roots derived from ordinary shoot cutting.

In *Arabidopsis thaliana*, the lateral root (LR) initiates from the primed LR founder cells in the xylem pole pericycle. After receiving particular signals, founder cells are activated and cell division is induced. The primordial LR begins to form and develops into the LR (Malamy and Benfey, 1997). Auxin plays an import role in this process (Benková et al., 2003; Dubrovsky et al., 2011; Sukumar et al., 2013; Villacorta-Martín et al., 2015). The polar transport of auxin from root tip to the aboveground part affects LR initiation, while auxin transport in the opposite direction influences LR germination (Reed et al., 1998; Casimiro et al., 2001). Many external factors affect LR initiation and growth by altering auxin distribution and polar transport, including gravity, bending, and mechanical stimulation (Mullen and Hangarter, 2003; De-Smet et al., 2007; Lucas et al., 2008). LR number decreased by ~90% in *transport inhibitor response (tir) 1* and *tir1/auxin-signaling f-box* (*afb*)*2/afb3* triple mutants, indicating important roles for auxin receptor and auxin signal transduction in LR development (Kepinski and Leyser, 2005; Péreztorres et al., 2008). De novo organogenesis of root primordium can be divided into two steps (Hu and Xu, 2016): the initial transition from regeneration-competent cells to root founder cells is accelerated by auxin-induced upregulation of *WUSCHEL-RELATED HOMEOBOX* (*WOX*)*11* and *WOX12*, after which WOX5/7 and its positive regulator WOX11/12 regulate root primordium initiation in *de novo* root organogenesis. These results suggest that early stages of root initiation are tightly regulated at the physiological as well as the genetic level. Most previous studies have focused on AR derived from the lower end of the stem, and it remains unclear how the stem apex of raspberries change from negative to positive geotropism and initiates rooting.

To address this issue, we analyzed gene expression in the stem apex during the early stages of adventitious rooting in primocane (i.e., the stem of the current season's growth) and floricane by Illumina HiSeq4000 analysis. We identified differentially expressed genes (DEGs) that may be involved in the regulation of AR formation in *Rubus*. Quantitative real-time (qRT)-PCR analysis was performed to validate the expression of some DEGs. Comparing gene expression patterns at three developmental stages of stem apex provided important insight into the molecular mechanisms of AR formation, for which we propose a model.

## Materials and methods

### Plant material

**Figure 1:**
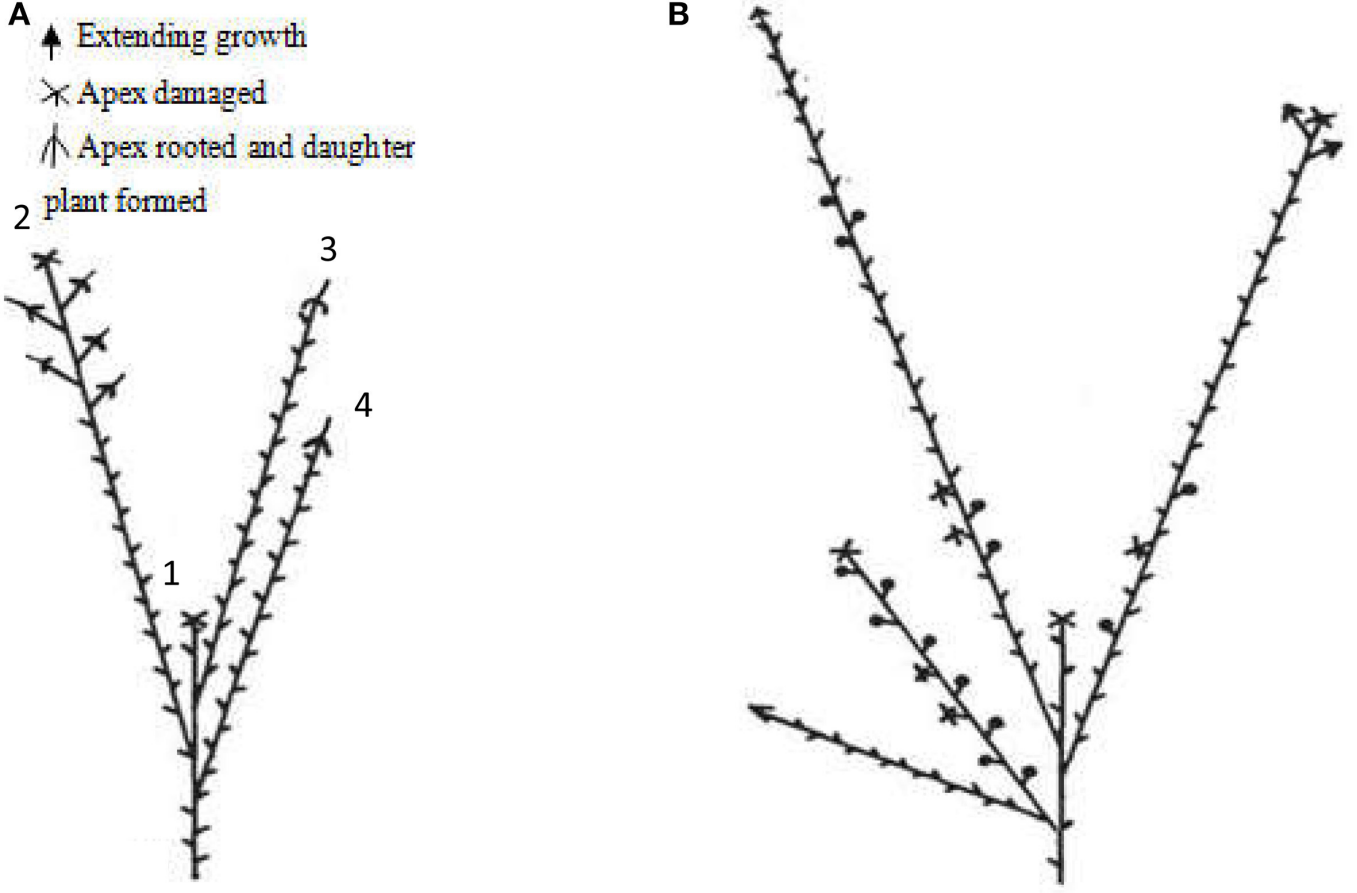
Diagrams of typical late season stems of “Sijihong” red raspberry (modified from Heslop-Harrison, 1959). The state of the buds is as indicated in the key. **(A)** Characteristics of primocane after terminal bud was removed (stem of the current season's growth). Shoot 2, 3, and 4 are newly induced lateral branches after the terminal bud 1 was cut off; **(B)** A 2-year-old floricane, terminal bud was cut during its first growth season.

**Figure 2:**
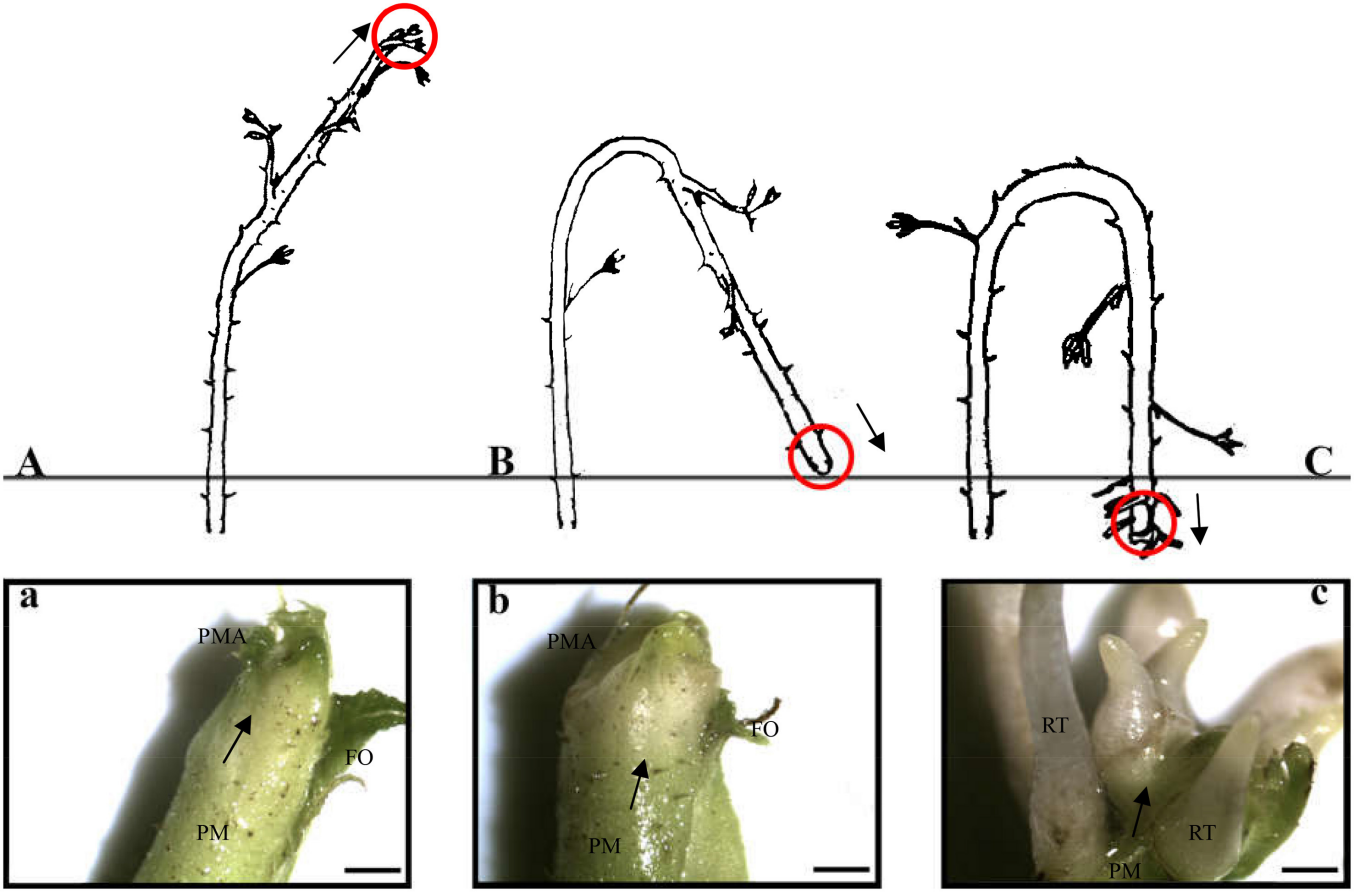
Three rooting developmental stages of *Rubus* spp. stem apex (modified from Heslop-Harrison, 1959. Panels **(a–c)** are enlarged views of the parts circled in red in panels **(A–C)**, respectively. **(A,a)** SAP1, foliage on the tapering apex remains active; **(B,b)** SAP2, foliage on the apex withers, apex becomes blunt and its diameter increased dramatically; **(C,c)** SAP3, the apex becomes chlorotic and short roots began to form. Black arrows in above panels show axis orientations. PM, primocane; PMA, primocane apex; FO, foliage; RT, root. Scale bars in a, b, and c = 2.0 mm.

Red raspberry (Sijihong), which exhibits the typical spontaneous rooting from the stem apex of primocane, was used in the present study. The experimental orchard was located in Siping City, Jilin Province, China. At the start of spring growth, there are two classes of stem—primocanes and floricanes—that will develop during that season (Bailey and Howard, 1941). Primocanes are the annual main stems; these are sterile, and apex removal will induce the formation of three or more new sterile primocanes (Figure 1A). Floricanes bear flowers and fruits, and apex removal will induce the formation of more floricanes that bear inflorescences either on the floricane itself or on their lateral branches (Figure 1B). By the end of summer, the height of primocanes may be more than 1 m long, and the apex begins to exhibit geotropism before subsequently differentiating. The sampling date of primocane is divided into the following three stages according to morphological observations (Figure 2): SAP1 (stage 1, during which foliage on the tapering apex remains active); SAP2 (stage 2, when apex foliage withers, the apex becomes blunt, and its diameter increases dramatically); and SAP3 (stage 3, when the apex becomes chlorotic and short roots begin to form). Primocane samples (0.5 cm at stem apex) were collected between 9:00 and 10:00 a.m. on July 10, August 10, and September 10. At the same times, three floricane samples (SAF1, SAF2, and SAF3, 0.5 cm at stem apex) were collected as control materials (Figure 2B). There were three biological replicates for each primocane and floricane sample. The samples were immediately frozen in liquid nitrogen and stored at −80°C until use.

### RNA extraction, library construction, and sequencing

In order to investigate global gene expression changes in the transcriptome during AR of the apex, we constructed 18 Digital Gene Expression (DGE) profiling libraries using the above-mentioned samples. Total RNA was extracted from the dissected tissue using the RNA Easyspin Isolation System (Aidlab Biotech, Beijing, China). RNA was dissolved in diethylpyrocarbonate-treated water and stored at −70°C until used for next-generation sequencing and quantitative real-time (qRT)-PCR validation. A total of 8 μg of total RNA was treated with DNase I and subjected to oligo (dT) magnetic bead adsorption to purify mRNA, which was fragmented by mixing with fragmentation buffer; the fragments were used for cDNA synthesis. Short fragments were purified and dissolved in elution buffer for end reparation and adenine addition, and then connected with adapters; suitable fragments were selected for PCR amplification. The Agilent 2100 Bioanalyzer (Agilent Technologies, Santa Clara, CA, USA) and ABI Step One Plus Real-Time PCR System (Applied Biosystems, Foster City, CA, USA) were used for quantitative analysis and validation of sample libraries, and all 18 libraries were sequenced using Illumina HiSeq 4000 (San Diego, CA, USA) (Cheng et al., 2015). All raw transcriptome data were deposited in the Sequence Read Archive (https://www.ncbi.nlm.nih.gov/SRA, accessions Number: SRP105309).

### Data analysis and mapping of DGE tags

Sequencing reads of low quality that were adaptor-polluted or had a high content of unknown base (N) reads were filtered. *De novo* assembly with clean reads was carried out to obtain the final unigenes, which were annotated by querying against National Center for Biotechnology Information (NCBI) nucleotide and protein (NR) databases and Clusters of Orthologous Groups, Kyoto Encyclopedia of Genes and Genomes (KEGG), and SwissProt databases (Altschul et al., 1990). Blast2GO with NRwas used for Gene Ontology (GO) annotation (Conesa et al., 2005), and InterProScan5 was used for InterPro annotation (Quevillon et al., 2005). Unigenes that could not be matched to any database mentioned above were identified by ESTScan with BLAST-predicted coding DNA sequences as a model (Yu et al., 2016). In total, six pairs of DGE profiles of different sample libraries (SAP1 vs. SAF1, SAP2 vs. SAF2, SAP3 vs. SAF3, SAP2 vs. SAP1, SAP3 vs. SAP2, SAP3 vs. SAP1, where the former was used as control and the latter as the experimental group) were compared to assess gene expression changes during AR formation in *Rubus*. A strict algorithm for identifying DEGs between two samples—the false discovery rate (FDR)—was used to determine the threshold *P*-value in multiple tests and analyses. FDR ≤ 0.001 and |log_2_Ratio| ≥ 1 were used as thresholds to identify DEGs. The possible functions of DEGs were determined by searching the GO database (http://www.geneontology.org/). Web Gene Ontology Annotation Plot (WEGO) was also used for GO classification of genes identified in each DGE library (Ye et al., 2006). To further characterize gene function, pathway enrichment analysis of the DGE results was performed by BLAST searches of the KEGG database (http://www.kegg.jp/kegg/). Cluster analysis of gene expression patterns was performed using Multi Experiment Viewer (http://mev.tm4.org/#/welcome). A *Q* ≤ 0.05 was selected as the threshold for significant enrichment of gene sets (Cheng et al., 2015).

### qRT-PCR analysis

DGE results were verified by qRT-PCR analysis. RNA samples used for qRT-PCR were identical to those used for DGE experiments. cDNA was synthesized using the PrimeScript RT Reagent kit (Takara Bio, Otsu, Japan) according to the manufacturer's instructions, and qRT-PCR was carried out using the SYBR Premix Ex Taq kit (Takara Bio) with gene-specific primers designed using Premier 6.0 software (Premier Biosoft International, Palo Alto, CA, USA) and synthesized commercially (Invitrogen, Carlsbad, CA, USA); the sequences are shown in Supplemental Table 1. The reaction mixture (final volume: 20 μL) contained 1.0 μL cDNA template, 2.0 μL 10 × reaction buffer, 0.5 μL dNTPs (10 mM), 1.0 μL each primer (10 μM), 10.0 μL 2× SYBR Green Supermix (Takara Bio), and 0.2 μL rTaq DNA polymerase (5.0 U/μL; Takara Bio), and the reaction was carried out on a Rotor-Gene 2000 thermocycler (Corbett Research, Sydney, Australia). Samples were prepared in triplicate in each experiment, and each biological sample consisted of two technical repeats. PCR conditions were as follows: 10 min at 95°C, followed by 40 cycles of 15 s at 95°C and 1 min at 60°C. Amplification data were analyzed using real-time analysis software (Corbett Research). Relative expression levels of genes were calculated with the 2^−ΔΔCt^ method (Cheng et al., 2015).

## Results

### Illumina sequencing and sequence assembly

To detect global gene expression changes during AR formation of the apex, 18 DGE libraries were sequenced using the Illumina HiSeq 4000 platform, generating ~50 million raw reads for each library. After filtering the reads containing adapter sequences and unknown nucleotides as well as those of low quality, 268.19 million clean reads with 26.82 Gb nucleotides were obtained (Table 1). *De novo* assembly yielded 59,173 unigenes with a mean length of 1,017 bp for all 18 libraries. Clean reads were deposited in the NCBI Sequence Read Archive (SRR3741688). Sequence annotation based on seven different public nucleotide/protein databases yielded 46,673 (78.88%) annotated unigenes in at least one of the databases, with ~21% of unigenes unmapped in any existing database (Table 2).

**Table 1:** Categorization and abundance of reads.

**Library**	**Summary of sequencing reads after filtering**				**Quality metrics of transcripts**			**Quality metrics of unigenes**		
	**Total raw reads (Mb)**	**Total clean reads (Mb)**	**Total clean bases (Gb)**	**Clean reads ratio (%)**	**Total number**	**Total length**	**Mean length**	**Total number**	**Total length**	**Mean length**
SAP1	49.77	44.74	4.47	89.9	61,650	54,075,084	877	42,844	44,864,341	1,047
SAP2	52.03	44.09	4.41	84.73	47,420	39,981,798	843	34,027	33,794,948	993
SAP3	49.77	44.64	4.46	89.69	52,791	43,351,596	821	37,061	36,390,097	981
SAF1	49.77	44.17	4.42	88.75	60,367	53,049,205	878	42,442	44,280,139	1,043
SAF2	52.03	45.88	4.59	88.17	59,408	52,368,945	881	41,755	43,681,476	1,046
SAF3	49.77	44.67	4.47	89.75	48,191	40,416,189	838	34,350	34,131,259	993

**Table 2:** Summary of function annotation.

	**Total**	**Nr annotated**	**Nt annotated**	**Swissprot annotated**	**KEGG annotated**	**COG annotated**	**Interpro annotated**	**GO annotated**	Overall^*^
Number	59,173	42,517	43,337	29,744	32,310	17,355	31,974	1,895	46,673
Percentage (%)	100	71.85	73.24	50.27	54.60	29.33	54.03	3.20	78.88

*Asterisk represents the number of unigenes which has matched annotation in at least one functional database*.

### Global gene expression in the stem apex of primocanes and floricanes

**Figure 3:**
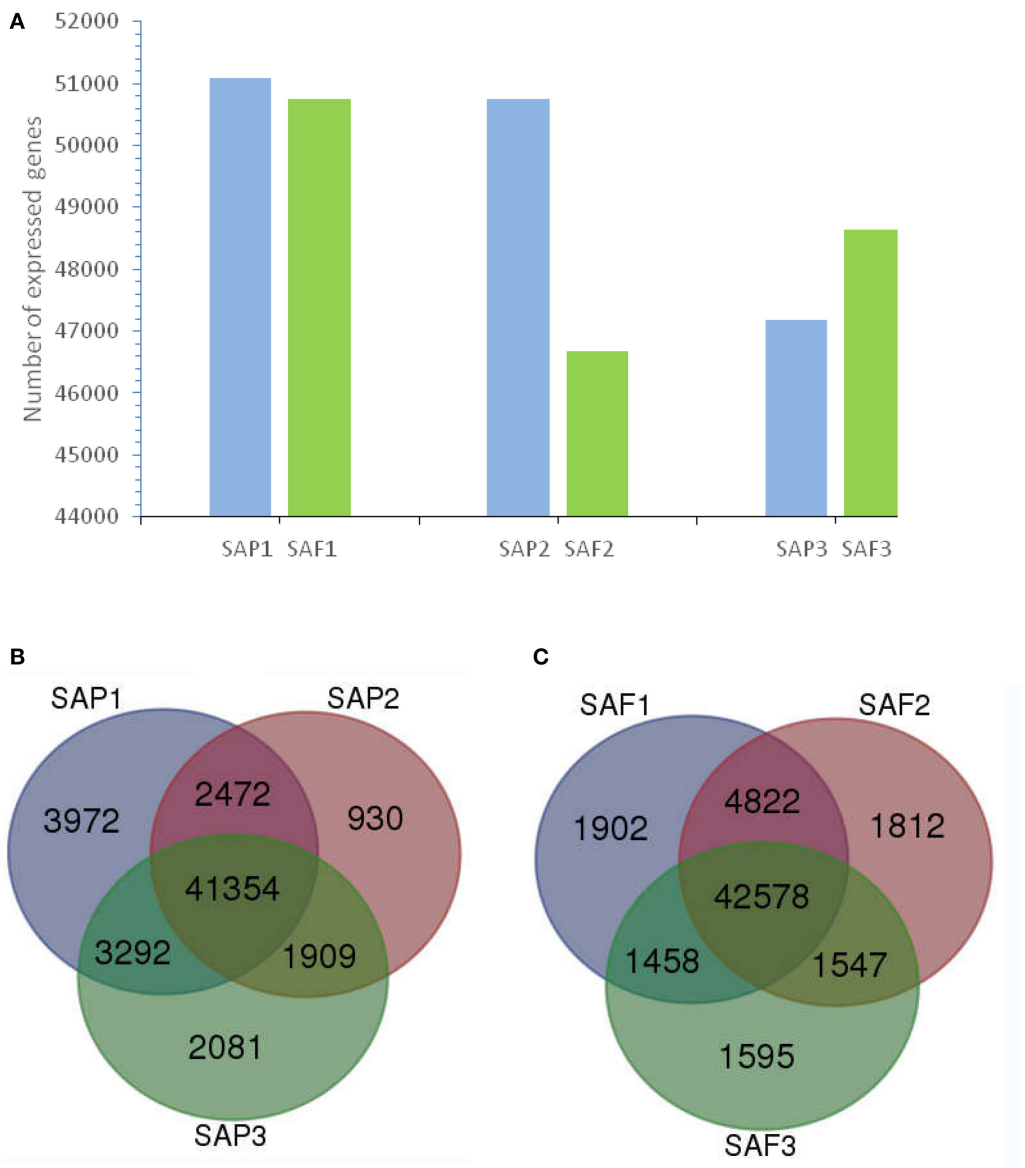
Transcriptome analysis of stem apex of primocanes and floricanes in *Rubus*. **(A)** Transcriptome sizes of stem apex of primocanes and floricanes at three developmental stages. **(B,C)** Venn diagram showing the overlaps between three stages of SAP and SAF. The number in parentheses after each stage designation is the total transcripts detected in that stage(s).

In total, 56,010 and 55,714 unigenes were expressed at the stem apex of primocanes and floricanes, respectively (Figure 3A). Of those expressed in primocanes, 41,354 were constitutively expressed at all three developmental stages, whereas 6,983 and 7,673 were specifically expressed in one and two developmental stages, respectively (Figure 3B). Of the unigenes expressed in floricanes, 42,578 were constitutively expressed at all three developmental stages, whereas 5,309 and 7,827 were specifically expressed in one and two developmental stages, respectively (Figure 3C). These changes in gene expression indicated that primocane apex rooting of *Rubus* is an involute biological process regulated by many genes. Compared to SAP2, SAP3, SAF1, SAF2, and SAF3, the greatest abundance of stage-specific transcripts (3,972) was detected in SAP1 of primocanes (Figures 3B,C), suggesting that a large number of specific genes is needed to coordinate the complex process of stem extension. Compared to SAP1, SAP3, SAF1, SAF2, and SAF3, 930 stage-specific transcripts were detected in SAP2 of primocanes (Figures 3B,C), indicating that transcripts regulating growth arrest and gravitropic deflection of primocane are expressed at this stage and initiate the growth transition from negative to positive gravitropism.

### DEGs in the stem apex of primocanes and floricanes

**Figure 4:**
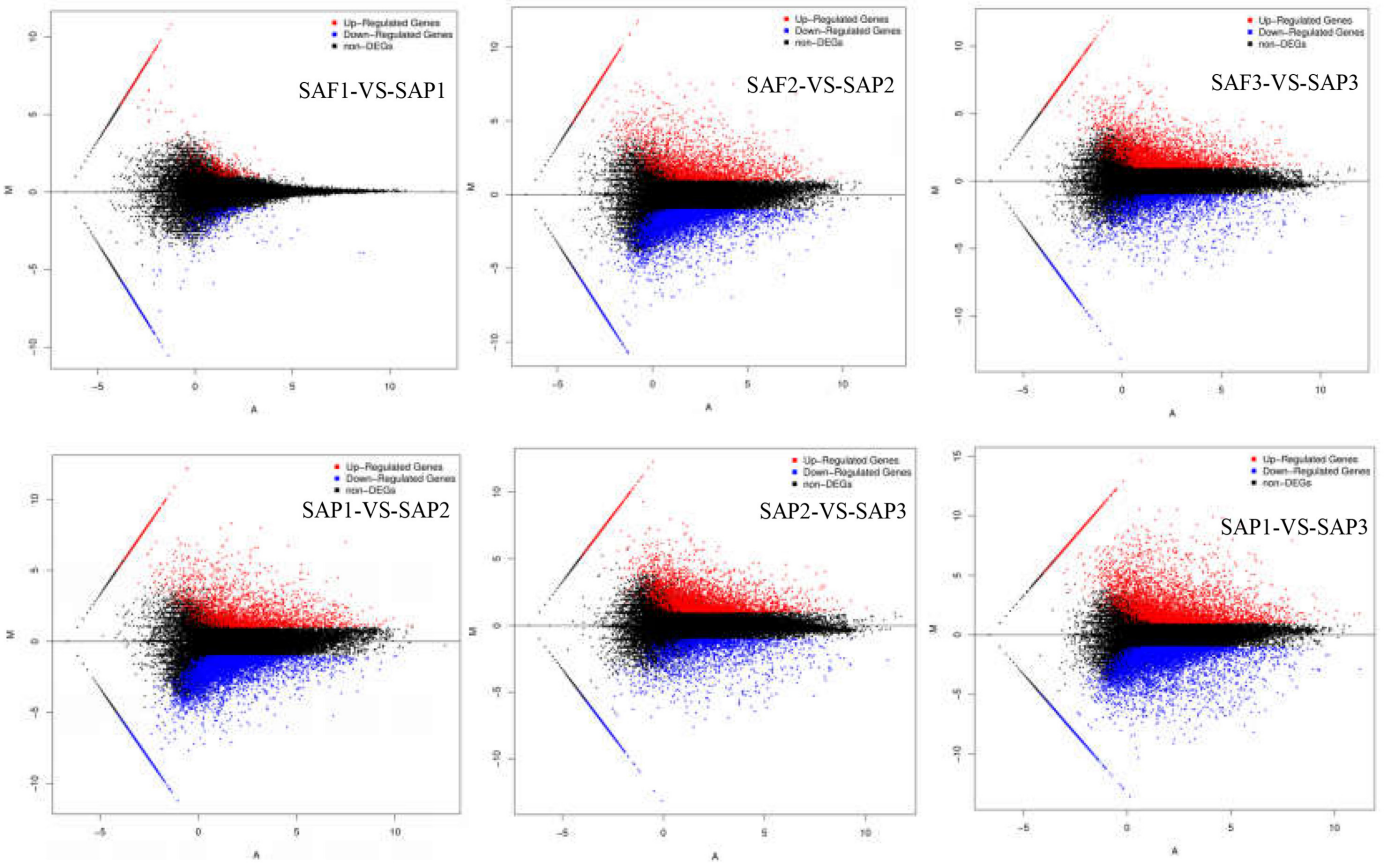
MA plot of DEGs. X axis represents value A (log2 transformed mean expression level). Y axis represents value M (log_2_ transformed fold change). Red points represent up regulated DEG. Blue points represent down regulated DEG. Black points represent non-DEGs.

FDR ≤ 0.001 and |log_2_Ratio| ≥1 were used as thresholds to identify DEGs (Figure 4; Table 3). Comparisons of primocane and floricanes at the same developmental time points revealed 7,346 (SAP2 vs. SAF2) and 4,110 (SAP3 vs. SAF3) DEGs; this number was much higher than the 498 DEGs in SAP1 vs. SAF1, in which case the number of up- and downregulated genes were similar. In contrast, in SAP2 vs. SAF2, there were more downregulated than upregulated DEGs, suggesting that foliar bud-related genes were silenced as the plant prepared for root differentiation. In SAP3 vs. SAF3, there were more upregulated than downregulated DEGs, possibly because root differentiation-related genes needed to be activated for the regulation of root development. Similarly, the analysis of floricanes at different time points revealed that most DEGs were downregulated in SAP2 vs. SAP1, while the majority of those in SAP3 vs. SAP2 were upregulated.

**Table 3:** Gene expression levels across different sample libraries.

**Differently expressed genes**	**SAF1-VS-SAP1**	**SAF2-VS-SAP2**	**SAF3-VS-SAP3**	**SAP1-VS-SAP2**	**SAP1-VS-SAP3**	**SAP2-VS- AP3**
Up-regulated	25	272	232	216	371	305
Highly up-regulated	199	409	565	429	1,109	596
Down-regulated	244	5,274	1,788	5,731	5,682	1,721
Highly down regulated	187	760	352	825	1,151	345
Total DEGs	498	7346	4,110	7,900	9,397	4,776
Not DEGs	53,366	45,174	46,577	44,775	43,423	4,6321
Total expression genes	54,251	53,689	52,169	53,929	55,080	52,038

*Highly up-regulated, log_2_Ratio ≥ 5 and FDR ≤ 0.01; Up-regulated, 5 ≥ log_2_Ratio ≥ 1 and FDR ≤ 0.01; Not DEGs, −1 ≤ log_2_Ratio ≤ 1 and FDR ≥ 0.01; Down-regulated, −5 ≤ log_2_Ratio ≤ −1 and FDR ≤ 0.01; Highly down-regulated, log_2_Ratio ≤ −5 and FDR ≤ 0.01*.

### Analysis of DEG clustering and pathway enrichment

**Figure 5:**
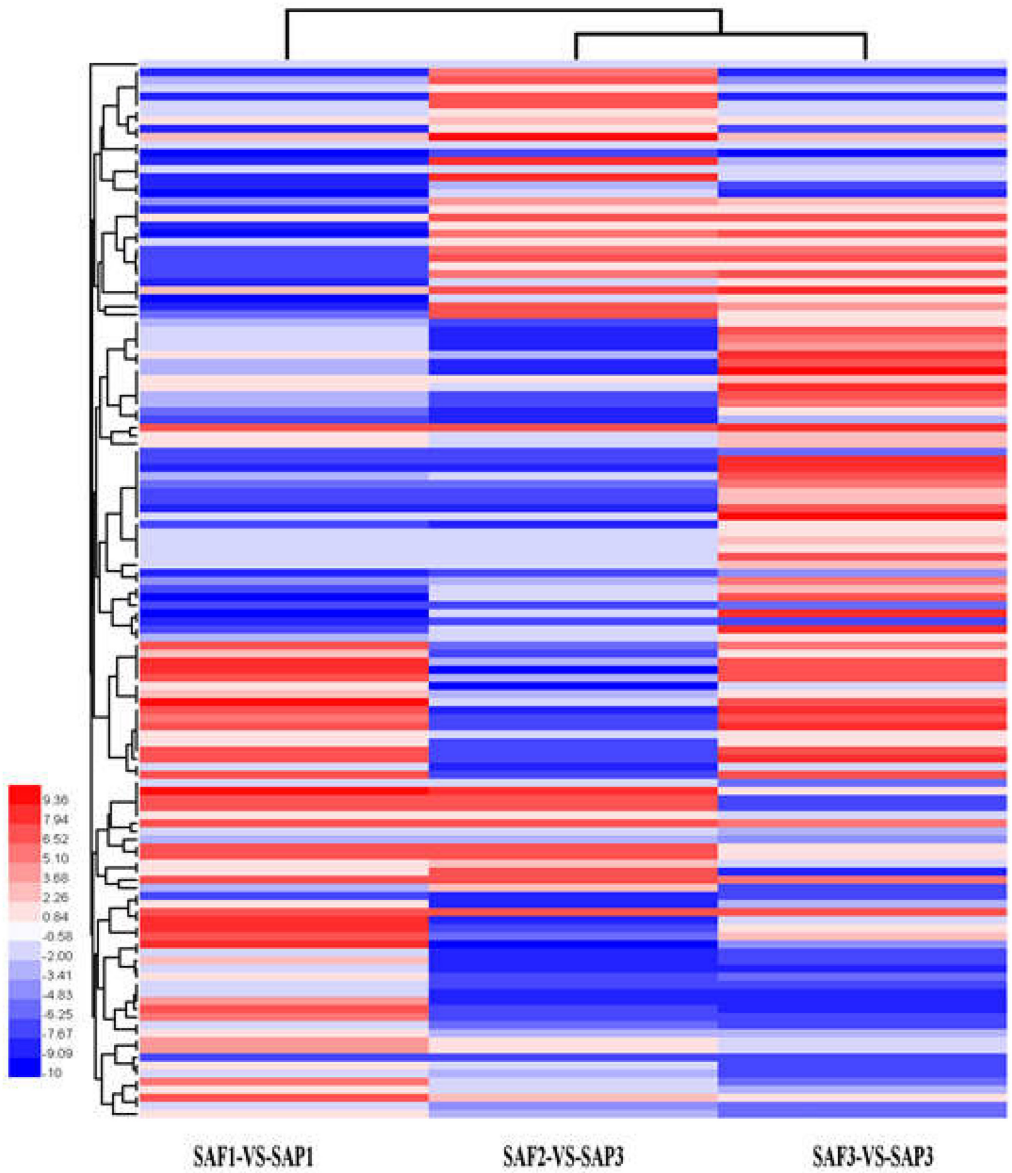
Hierarchical clustering (HCE) analysis of differentially expressed transcripts between stem apex of primocane and floricane at three developmental stages (SAP1-VS-SAF1, SAP2-VS-SAF2, and SAP3-VS-SAF3, “a” was the control and “b” was experimental group in “a-VS-b”). The clusters from A to H indicate the eight major clusters resulting from HCE analysis. Each line refers to data from one gene. The color bar represents the log_10_RPKM and ranges from blue (low expression) to red (high expression).

**Figure 6:**
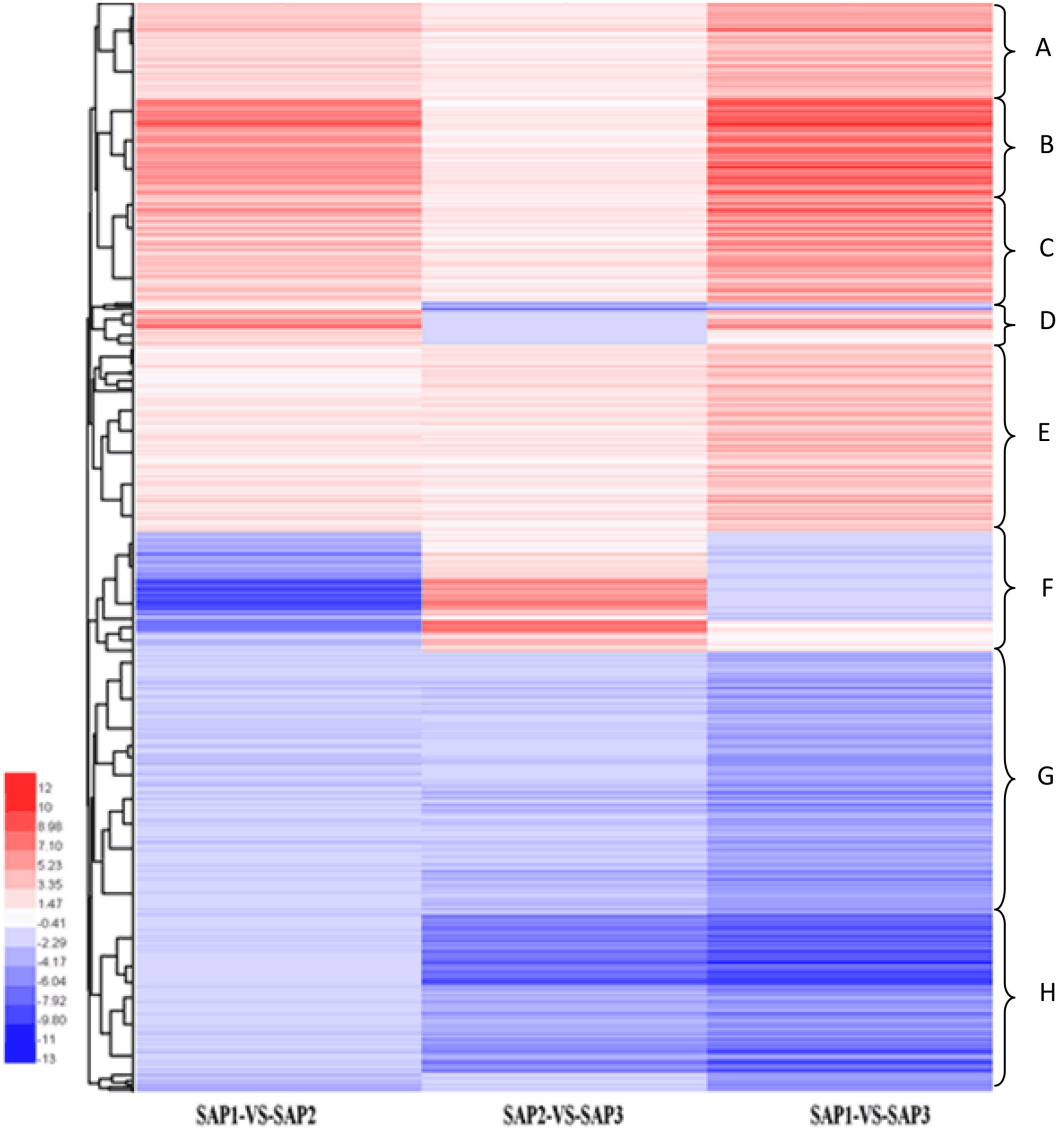
Hierarchical cluster analysis of DEGs between different developmental stages for stem apex of primocane and floricane (SAP1 vs. SAP2, SAP2 vs. SAP3, SAP1 vs. SAP3, “a” was the control and “b” was experimental group in “a vs. b”). Each line refers to data from one gene. The color bar represents the log_10_RPKM and ranges from green (low expression) to red (high expression). The clusters from A to H indicate the eight major clusters resulting from HCE analysis.

Hierarchical clustering analysis of differentially expressed transcripts in three SAF vs. SAP paired comparisons revealed that only 131 transcripts were common to all three developmental stages (Figure 5), indicating that stage-specific transcripts were likely responsible for the regulation of AR differentiation. The analysis of DEGs in SAP1 vs. SAP2, SAP2 vs. SAP3, and SAP1 vs. SAP3 paired comparisons revealed 1,365 transcripts common to all three developmental stages in primocane, which grouped into eight categories (Figure 6). Apart from categories D and F, the other six categories (comprising 1,162 DEGs) showed similar trends of up- or down-regulation in SAP1 vs. SAP2 and SAP2 vs. SAP3 paired comparisons, indicating that AR induction and differentiation are closely and sequentially regulated at the level of gene expression.

**Figure 7:**
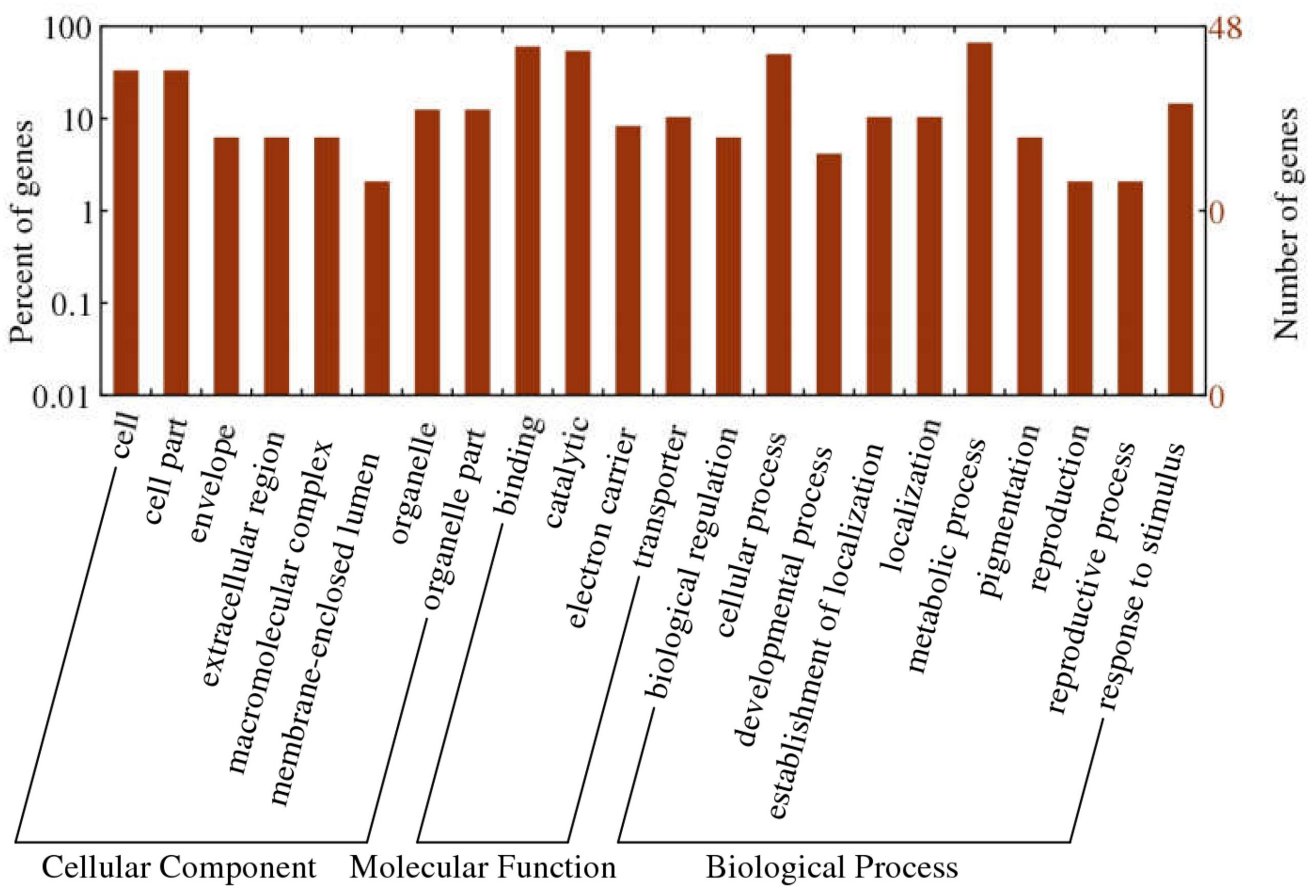
Gene Ontology (GO) categories of common DEGs in SAF-VS-SAP paired comparisons.

To obtain functional information about the 1,365 common DEGs, we carried out a literature search and annotated biochemical and biological functions according to the WEGO database. Results of GO functional enrichment are shown in Figure 7. For cellular position, the categories with considerable enrichment and largest number of DEGs were cell (33.3%) and cell part (33.3%). For biological process, the categories with the greatest enrichment and largest number of DEGs were metabolic process (66.7%), cellular process (50.0%), and response to stimulus (14.6%). For molecular function, binding (60.4%) and catalytic activity (54.2%) were the most highly represented categories.

Stage 2 is the transition period from bud development to root formation; as such, DEGs at this stage are presumed to play an important role in determining the developmental course of *Rubus* primocane apex. We performed a KEGG pathway classification and functional enrichment analysis for DEGs identified in SAP1 vs. SAP2. A total of 12 pathways were significantly enriched, with a *Q* < 0.05 (Table 4). These DEGs were associated with flavonoid biosynthesis; circadian rhythm—plant; biosynthesis of secondary metabolites; stilbenoid, diarylheptanoid, and gingerol biosynthesis; cyanoamino acid metabolism; sesquiterpenoid and triterpenoid biosynthesis; porphyrin and chlorophyll metabolism; photosynthesis—antenna proteins; limonene and pinene degradation; plant hormone signal transduction; phenylpropanoid biosynthesis; and ascorbate and aldarate metabolism. These results suggest that changes in the expression of genes related to metabolism, environmental adaptation, and signal transduction are involved in the transition from bud development to root formation at the primocane apex of *Rubus*.

**Table 4:** Significant enriched pathways in SAP1-VS-SAP2 paired comparison of *Rubus*.

**No**.	**Pathway**	**DEGs genes with pathway annotation (6157)**	**All genes with pathway annotation (32310)**	***P*-value**	***Q*-value**	**Pathway ID**	**Level 1**	**Level 2**
1	Flavonoid biosynthesis	96 (1.56%)	237 (0.73%)	1.60E-14	2.14E-12	ko00941	Metabolism	Biosynthesis of other secondary metabolites
2	Circadian rhythm - plant	94 (1.53%)	269 (0.83%)	5.29E-10	3.54E-08	ko04712	Organismal Systems	Environmental adaptation
3	Biosynthesis of secondary metabolites	916 (14.88%)	4123 (12.76%)	2.93E-08	1.31E-06	ko01110	Metabolism	Global and overview maps
4	Stilbenoid, diarylheptanoid and gingerol biosynthesis	81 (1.32%)	242 (0.75%)	6.96E-08	2.33E-06	ko00945	Metabolism	Biosynthesis of other secondary metabolites
5	Cyanoamino acid metabolism	105 (1.71%)	349 (1.08%)	4.28E-07	1.15E-05	ko00460	Metabolism	Metabolism of other amino acids
6	Sesquiterpenoid and triterpenoid biosynthesis	40 (0.65%)	105 (0.32%)	4.16E-06	9.30E-05	ko00909	Metabolism	Metabolism of terpenoids and polyketides
7	Porphyrin and chlorophyll metabolism	68 (1.1%)	213 (0.66%)	4.87E-06	9.33E-05	ko00860	Metabolism	Metabolism of cofactors and vitamins
8	Photosynthesis - antenna proteins	18 (0.29%)	36 (0.11%)	2.80E-05	4.69E-04	ko00196	Metabolism	Energy metabolism
9	Limonene and pinene degradation	48 (0.78%)	146 (0.45%)	4.95E-05	7.37E-04	ko00903	Metabolism	Metabolism of terpenoids and polyketides
10	Plant hormone signal transduction	251 (4.08%)	1074 (3.32%)	0.000201	2.69E-03	ko04075	Environmental Information Processing	Signal transduction
11	Phenylpropanoid biosynthesis	194 (3.15%)	813 (2.52%)	0.000336	4.10E-03	ko00940	Metabolism	Biosynthesis of other secondary metabolites
12	Ascorbate and aldarate metabolism	59 (0.96%)	212 (0.66%)	0.001173	1.31E-02	ko00053	Metabolism	Carbohydrate metabolism

### DEGs associated with primocane apex rooting in *Rubus*

Based on prior knowledge of their putative involvement in shoot and root development and enriched KEGG pathways of genes that were differentially expressed between primocane and floricane stem apices at three development stages, several subgroups were determined to be related to root induction and differentiation (Table 5) in SAP1-VS-SAP2 and SAP2-VS-SAP3 paired comparisons of *Rubus*. In the former, we identified 24 unigenes involved in the regulation of root induction and differentiation at the primocanes apex, whose expression changed significantly; these included *PHYTOCHROME* (*PHY*)*B* (CL1524.Contig3_All, −1.21), *EARLY FLOWERING* (*ELF*)*3* (CL2434.Contig1_All, −1.71), *ARABIDOPSIS PSEUDO-RESPONSE REGULATOR* (*APRR*)*5* (CL5214.Contig6_All, −10.36), *CYCLING DOF FACTOR* (*CDF*)*1* (Unigene16183_All, −3.45), and *HECATE* (*HEC*)*2* (Unigene21192_All, 3.22) in the circadian rhythm–plant pathway; and *1-AMINOCYCLOPROPANE-1-CARBOXYLATE SYNTHASE* (*ACS*) (Unigene20575_All, 8.07), *AUXIN RESPONSE FACTOR* (*ARF*) (CL1090.Contig5_All, −9.79; CL2444.Contig1_All, −7.96; CL1556.Contig5_All, −7.51; Unigene20581_All, −3.30; CL5079.Contig1_All, −3.16; CL1556.Contig2_All, −3.17; Unigene15505_All, 4.58.), *THREONINE-PROTEIN KINASE CTR1* (*CTR1*) (Unigene24307_All, 7.91), and *ETHYLENE-RESPONSIVE TRANSCRIPTION FACTOR* (*ERF*)*1* (Unigene13079_All, 4.00) in the cysteine and methionine metabolism and plant hormone signal transduction pathways. This latter suggested that ethylene biosynthesis and ethylene and auxin signaling play important roles in regulating shoot elongation arrest and initiation of root differentiation. We also identified a set of shoot and root development-related genes, including *SHOOT MERISTEMLESS* (*STM*) (CL6648.Contig1_All, 1.16; CL6648.Contig3_All, 1.84) related to shoot growth, and *SHOOT GRAVITROPISM* (*SGR*)*5* (Unigene12164_All, −1.00), which is responsible for changes in shoot gravitropism during the transition from bud to root; seven DEGs were involved in regulating root primordium, root phototropism, lateral root primordium (LRP), and root cap, including CL6906.Contig3_All (−1.25), Unigene15061_All (−2.63), Unigene16263_All (2.99), Unigene17114_All (5.13), Unigene20360_All (−1.47), Unigene331_All (−1.49), and Unigene7065_All (4.53). Taken together, their up/downregulation in the SAP1 vs. SAP2 paired comparison was presumed to be beneficial for stem elongation growth arrest, stem gravitropic deflection, and root primordium differentiation.

**Table 5:** Selected DEGs that may be beneficial for primocane apex rooting in SAP1-VS-SAP2 and SAP2-VS-SAP3 paired comparisons of *Rubus*.

**Function group**	**GeneID**	**log_2_FoldChange(SAF-VS-SAP)**			**log_2_FoldChange (SAP-VS-SAP)**			**Gene annotation**
		**SAF1-VS-SAP1**	**SAF2-VS-SAP2**	**SAF3-VS-SAP3**	**SAP1-VS-SAP2**	**SAP2-VS-SAP3**	**SAP1-VS-SAP3**	
Circadian rhythm - plant	CL1524.Contig3_All	−0.10	−1.29^*^	−0.06	−1.21^*^	−0.26	−1.48^*^	*PHYB, PHYTOCHROME B*
	CL2434.Contig1_All	−0.18	−1.45^*^	−0.30	−1.71^*^	−0.02	−1.72^*^	*ELF3, EARLY FLOWERING 3*
	CL5214.Contig6_All	−0.15	−9.79^**^	−4.39	−10.36^**^		−10.36^**^	*APRR5, TWO-COMPONENT RESPONSE REGULATOR APRR5*
	Unigene16183_All	0.04	−3.40^**^	0.18	−3.45^**^	0.16	−3.30^**^	*CDF1, CYCLING DOF FACTOR 1*
	Unigene21192_All	−0.25	3.05^*^	−3.88^**^	3.22^*^	−4.40^**^	−1.18	*HEC2, HECATE 2*
Cysteine and methionine metabolism	Unigene20575_All	−4.00	8.07^*^	−0.07	8.07^*^	−0.83	7.24^*^	*ACS, 1-AMINOCYCLOPROPANE-1-CARBOXYLATE SYNTHASE*
Plant hormone signal transduction	CL1090.Contig5_All	−0.38	−9.86^**^	0.95	−9.79^**^	5.91^**^	−3.88^**^	*ARF1, AUXIN RESPONSE FACTOR 1*
	CL2444.Contig1_All	−0.07	−8.07^**^	−4.58	−7.96^**^		−7.96^**^	*ARF18*
	CL1556.Contig5_All	0.14	−7.09^**^	−5.17^**^	−7.51^**^		−7.51^**^	*ARF7*
	Unigene20581_All	0.7	−2.81^*^	−0.1	−3.30^**^	1.00	−2.30^*^	*ARF7*
	CL5079.Contig1_All	−0.07	−2.86^*^	−1.46	−3.16^**^	−1.58	−4.74^**^	*ARF23*
	CL1556.Contig2_All	0.42	−3.19^**^	0	−3.17^**^	0.70	−2.47^*^	*ARF7*
	Unigene24307_All		3.05^**^	4.81^**^	7.91^**^	−3.10^**^	4.81	*CTR1, THREONINE-PROTEIN KINASE CTR1*
	Unigene13079_All	−0.57	4.03^**^	−0.53	4.00^**^	−0.76	3.23^**^	*ERF1, ETHYLENE-RESPONSIVE TRANSCRIPTION FACTOR1*
	Unigene15505_All		4.58	9.12^**^	4.58	4.53^**^	9.12^**^	*ARF7*
	CL5715.Contig2_All	−0.02	−1.82^*^	4.17^**^	−1.49	4.03^**^	2.54^*^	*CTR1*
	Unigene1657_All	−1.58	−5.95	7.92^**^	−3.70	7.92^**^	4.22^**^	*EIN3, ETHYLENE-INSENSITIVE3*
Shoot and root development	CL6648.Contig1_All	−0.12	1.11^*^	−0.25	1.16^*^	−0.24	0.92	*STM, SHOOT MERISTEMLESS*
	CL6648.Contig3_All	0.05	1.51^*^	−0.44	1.84^*^	−0.88	0.96	*STM*
	CL6906.Contig3_All	−0.03	−1.30^*^	0.48	−1.25^*^	0.59	−0.65	*RPD1, ROOT PRIMORDIUM DEFECTIVE 1*
	Unigene12164_All	−0.27	−1.14^*^	−1.76^*^	−1.00^*^	−1.57^*^	−2.57^*^	*SGR5, SHOOT GRAVITROPISM 5*
	Unigene15061_All	0.08	−2.99^*^	−3.81	−2.63^*^	−4.86	−7.49^**^	*RPT3, ROOT PHOTOTROPISM 3*
	Unigene16263_All	−0.31	2.51^*^	0.67	2.99^*^	0.90	3.89^**^	*LRP, LATERAL ROOT PRIMORDIUM*
	Unigene17114_All	3.81	4.61^**^	1.94^*^	5.13^**^	1.53*	6.65^**^	*RC, ROOT CAP*
	Unigene20360_All	0.47	−1.65^*^	−0.15	−1.47^*^	−0.10	−1.57^*^	*RPD1*
	Unigene331_All	−0.14	−1.59^*^	0.04	−1.49^*^	0.38	−1.11^*^	*RPD1*
	Unigene7065_All	−1.00^*^	7.34^**^	3.96^**^	4.53^**^	3.10^**^	7.63^**^	*RC*
	Unigene811_All	−0.27	−0.96	−1.99^*^	−0.61	−2.07^*^	−2.68^*^	*RC*
	CL1554.Contig3_All	−0.33	0.07	−1.76^*^	0.38	−1.65^*^	−1.27^*^	*RPT2*
	CL1554.Contig1_All		0.04	−1.62^*^	0.11	−1.60^*^	−1.50^*^	*RPT2*
	CL1554.Contig2_All	0.39	−0.15	−1.22^*^	−0.30	−1.26^*^	−1.57^*^	*RPT2*
	CL4872.Contig3_All	1.41^*^	1.85	2.58^*^	−1.76	3.27^**^	1.51	*RHD3, ROOT HAIR DEFECTIVE 3*
	CL6648.Contig2_All	−0.63	1.31		0.09	−7.65^**^	−7.56^**^	*STM*
	Unigene10691_All	−0.01	−0.09	−4.48^**^	0.10	−4.70^**^	−4.61^**^	*SGR5*
	Unigene10761_All	−0.06	0.85	1.18^*^	0.87	1.22^*^	2.09	*AIR12, AUXIN INDUCED IN ROOTS 12*

*Asterisk represent DEGs with a threshold of FDR ≤ 0.001 and |log_2_Ratio| ≥ 1, and double asterisks represent highly regulated DEGs with a threshold of FDR ≤ 0.001 and |log_2_Ratio| ≥ 3*.

Stage 3 is the major period in which the root primordium differentiates from stem apex and the root extends. In the SAP2 vs. SAP3 paired comparison, we identified 13 DEGs specific to this period that may facilitate rooting. In the plant hormone signal transduction pathway, two DEGs encoding ARFs (CL1090.Contig5_All and Unigene15505_All) were highly upregulated (5.91 and 4.53 fold, respectively); at the same time, three highly DEGs encoding *CTR1* (Unigene24307_All, −3.10; CL5715.Contig2_All, 4.03) and one encoding *ETHYLENE-INSENSITIVE* (*EIN*)*3* (Unigene1657_All, 7.92) were identified. Auxin and ethylene signaling pathways were more active in stage 3 than in stage 2. A set of root development-related DEGs were identified, including three DEGs encoding *ROOT CAP* (*RC*) (Unigene17114_All, 1.53; Unigene7065_All, 3.10; Unigene811_All, −2.07), three encoding *ROOT PHOTOTROPISM* (*RPT*)*2* (CL1554.Contig3_All, −1.65; CL1554.Contig1_All, −1.60; CL1554.Contig2_All, −1.26), and one each encoding *ROOT HAIR DEFECTIVE* (*RHD*)*3* (CL4872.Contig3_All, 3.27) and *AUXIN-INDUCED IN ROOT CULTURES* (*AIR*)*12* (Unigene10761_All, 1.22); their up/downregulation in SAP2 vs. SAP3 paired comparisons was presumed to facilitate to root cap and negative root phototropism formation.

### DEGs validated by qRT-PCR

**Figure 8:**
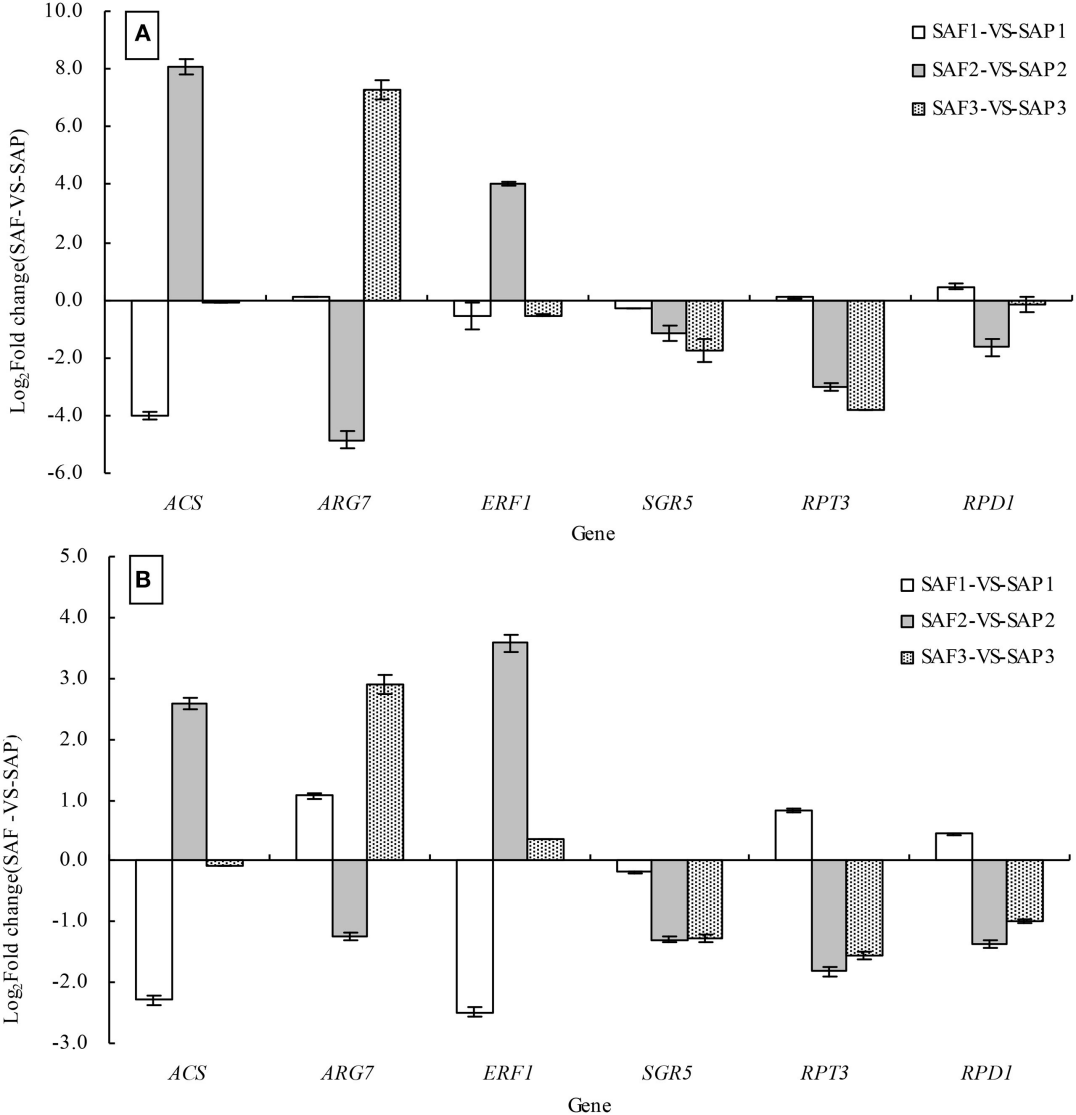
Validation of DEGs by qRT-PCR analysis. Note: the relative expression levels of six differential expressed genes in three development stages of primocanes and floricanes were obtained by RNA-seq using RPKM method **(A)** and by qRT-PCR using the 2^−ΔΔCT^ method **(B)**. Bars represent mean ± SE (*n* = 6).

To validate the DEGs identified in the SAP1 vs. SAF1, SAP2 vs. SAF2, and SAP3 vs. SAF3 paired comparisons, six genes were selected for validation by qRT-PCR analysis, including *ACS, ARG7* (*INDOLE-3-ACETIC ACID-INDUCED PROTEIN ARG7*), *ERF1, SGR5, RPT3*, and *RPD1* (*ROOT PRIMORDIUM DEFECTIVE 1*), all of which have been implicated in the regulation of root development. The expression of all six genes as determined by qRT-PCR was consistent with the DGE patterns (Figures 8A,B).

## Discussion

### Ethylene and auxin play essential roles in the regulation of *Rubus* primocane apex rooting

AR formation is a quantitative genetic trait regulated by both environmental (e.g., temperature, light, and relative humidity) and endogenous (e.g., levels of hormone, sugar, mineral salt, and other molecules) factors (Tang et al., 2016; Zerche et al., 2016). Hormones play an important role in this process as plants respond to the changing environment, and influence cell fate specification via regulation of downstream gene expression. This was confirmed by the results of the DEG pathway enrichment analysis, which showed that plant hormone signal transduction (ko04075) and cysteine and methionine metabolism (ko00270) were significantly enriched (Table 4).

Auxin is the principal phytohormone that initiates rooting and is critical for the first phase of AR formation (Ribeiro et al., 2016). ARF act as an important positive regulator of auxin signaling; two DEGs encoding ARFs were highly upregulated while six encoding ARFs were downregulated in AP2-VS-SAP3 and SAP1-VS-SAP2 paired comparisons, respectively, implying that auxin signaling tended to be activated in stage 3 and inactivated in stage 2. In *Arabidopsis*, auxin is a positive regulator of ethylene-mediated response in root growth, and ARF17, ARF6, and ARF8 act as negative or positive regulators of adventitious rooting (Rahman et al., 2001; Gutierrez et al., 2009). The upregulation of ARF-encoding DEGs (CL1090.Contig5_All, Unigene15505_All) in the SAP2 vs. SAP3 paired comparison is presumed to be beneficial for AR formation in stage 3, while their downregulation in the SAP1 vs. SAP2 paired comparison is expected to induce stem elongation arrest in stage 2. Aux/IAA acts as a negative regulator of auxin signaling. In accordance with the activation and inactivation of auxin signaling, highly downregulated DEGs (CL130.Contig7_All, −7.70) and upregulated DEGs encoding Aux/IAA (Unigene18450_All, 2.3) were also identified in the SAP1 vs. SAP2 and SAF2 vs. SAP3 paired comparisons, respectively. Thus, auxin may play an important role in regulating stem elongation in stage 2 and root differentiation in stage 3 by inhibiting and inducing the expression of *ARFs and AUX/IAA*.

Ethylene is thought to interact with auxin in the control of adventitious rooting in stems or stem cuttings (Da et al., 2013). ACS is a key enzyme in ethylene biosynthesis; a DEG (Unigene20575_All) encoding ACS was upregulated by 8.07 and 7.24 fold in the SAP1 vs. SAP2 and SAP1 vs. SAP3 paired comparisons, respectively, implying that ethylene biosynthesis was activated at stages 2 and 3. Wounding and other abiotic stress factors activate ethylene biosynthesis, and AR formation can be stimulated by ERFs (Druege et al., 2014, 2016). CTR1 is a negative regulator of the ethylene response pathway in *Arabidopsis* (Solano et al., 1998). Ethylene signaling was active at stages 2 and 3, as evidenced by the upregulation of positive regulators *ERF1* (Unigene13079_All) and *EIN3* (Unigene1657_All) and downregulation of *CTR1* (Unigene24307_All). Taken together, these results suggest that activation of ethylene biosynthesis via *ACS, EIN3*, and *ERF1* signaling contributes to the termination of bud development at stage 2, and subsequently induces AR formation at stage 3 in *Rubus*.

### The circadian clock is an important system controlling AR differentiation in *Rubus*

Stem elongation in *Rubus* is enhanced by long-day conditions (Sønsteby and Heide, 2009). The rooting process was previously thought to be a response to the shortening day length in late summer in European Rubi of the subgenus *Eubatus* (Heslop-Harrison, 1959). The three floricane samples were collected on July 10, August 10, and September 10, and our data suggest that rooting at primocane stem apex is regulated by photoperiod. The significant enrichment of the KEGG circadian rhythm–plant pathway implies that AR formation at the *Rubus* stem apex involves circadian rhythm-related genes. DEGs in the circadian rhythm—plant pathway included *PHYB, APRR5, ELF3*, and *CDF1*, all of which were downregulated in SAF2 vs. SAP2 and SAP1 vs. SAP2 paired comparisons, while *HEC2* expression showed the opposite trend. APRR5 is associated with the inhibition of leaf expansion in the *Arabidopsis* shade-avoidance response (Takase et al., 2013). The depressed expression of *HEC* showed pin-shaped inflorescences in *Arabidopsis thaliana*, suggesting that HEC genes are involved in auxin-mediated control of gynoecium patterning (Gremski et al., 2007). In rice, *PHYB* mutants exhibited reduced total leaf area per plant (Liu et al., 2012). ELF3 is a nuclear protein that is expressed rhythmically and interacts with PHYB to control plant development and flowering, and is an important component of the core circadian clock independent of light conditions. We found that *PHYB, APRR5, ELF3, CDF1*, and *HEC2* were highly expressed at stage 2, corresponding to the shortening of daylight time. PHYB along with ELF3 may regulate circadian rhythm, while PHYB and APRR5 may inhibit leaf growth and expansion at the stem apex and induce its final withering. The arrest in bud differentiation may create beneficial physiological conditions for root primordium differentiation. Thus, HEC2 may influence root induction and differentiation via auxin.

### Direct regulator of primocane apex rooting in raspberry

Roots play important roles in plant growth and development, including water and nutrient uptake and anchoring the plant in soil. In *Rubus*, AR formation implies the formation of new daughter plants. In *Arabidopsis thaliana, RPD1* is associated with the maintenance of active cell proliferation, and its mutation was found to impair axis formation and cellular patterning at early stages of root primordium development (Konishi and Sugiyama, 2006). Tropism induces root growth and determines the eventual vertical growth trajectory of plants. Roots exhibit a negative phototropic growth pattern in response to blue and white light, which is mediated by phytochrome, which may regulate positive phototropism in roots (Kiss et al., 2003). The loss-of-function mutant of *RPT2* revealed a role in phot1-induced phototropic response and stomatal opening of roots (Inada et al., 2004). *RHD3* may be regulated by auxin and ethylene, and itself regulates cell enlargement and root development (Wang et al., 1997). *AIR12* regulates the development of epidermal cells surrounding the emerging lateral root (Costa et al., 2016). RC acting as a gravity-sensing site can sense environmental cues to control the growth direction of the root tip as well as regulate root growth and protect internal cells (Wang et al., 2005; Suzuki et al., 2016). ARFs in auxin signaling are indispensable for root cap development (Wang et al., 2005). In the present study, DEGs encoding *RPD1, RC, LRP, RPT2, RPT3, AIR12*, and *RHD3* were identified in AP1-VS-SAP2 and SAP2-VS-SAP3 paired comparisons, and we concluded that their up- or downregulation facilitate axis formation and cellular patterning, cell enlargement, negative phototropic growth, stomatal opening, and gravitropic sensing and control growth direction of roots, thereby contributing to root differentiation and development in stages 2 and 3. Considering the potential regulatory roles of phytochrome, auxin, and ethylene in root phototropism, we speculate that there is crosstalk between the circadian rhythm–plant and plant hormone signal transduction pathways and root development.

### Model for regulation of rooting genes at the stem apex of *Rubus*

Accumulating evidence suggests reciprocal interactions between the circadian clock, metabolism, and stress signaling in the control of *Arabidopsis* growth (Müller et al., 2014). In sunflower (*Helianthus annuus* L.), gibberellins, auxin, and ethylene directly regulate light-mediated changes in shoot growth. The first two function as growth promoters, whereas ethylene is a growth inhibitor that probably interacts with gibberellins in controlling stem and leaf growth along the sunflower shoot (Kurepin and Pharis, 2014). Normal levels of abscisic acid (ABA) are important for shoot development and suppress ethylene synthesis in tomato (Lenoble and Sharp, 2004). Thus, ABA deficiency during shoot growth may be at least partly attributable to increased ethylene production.

**Figure 9:**
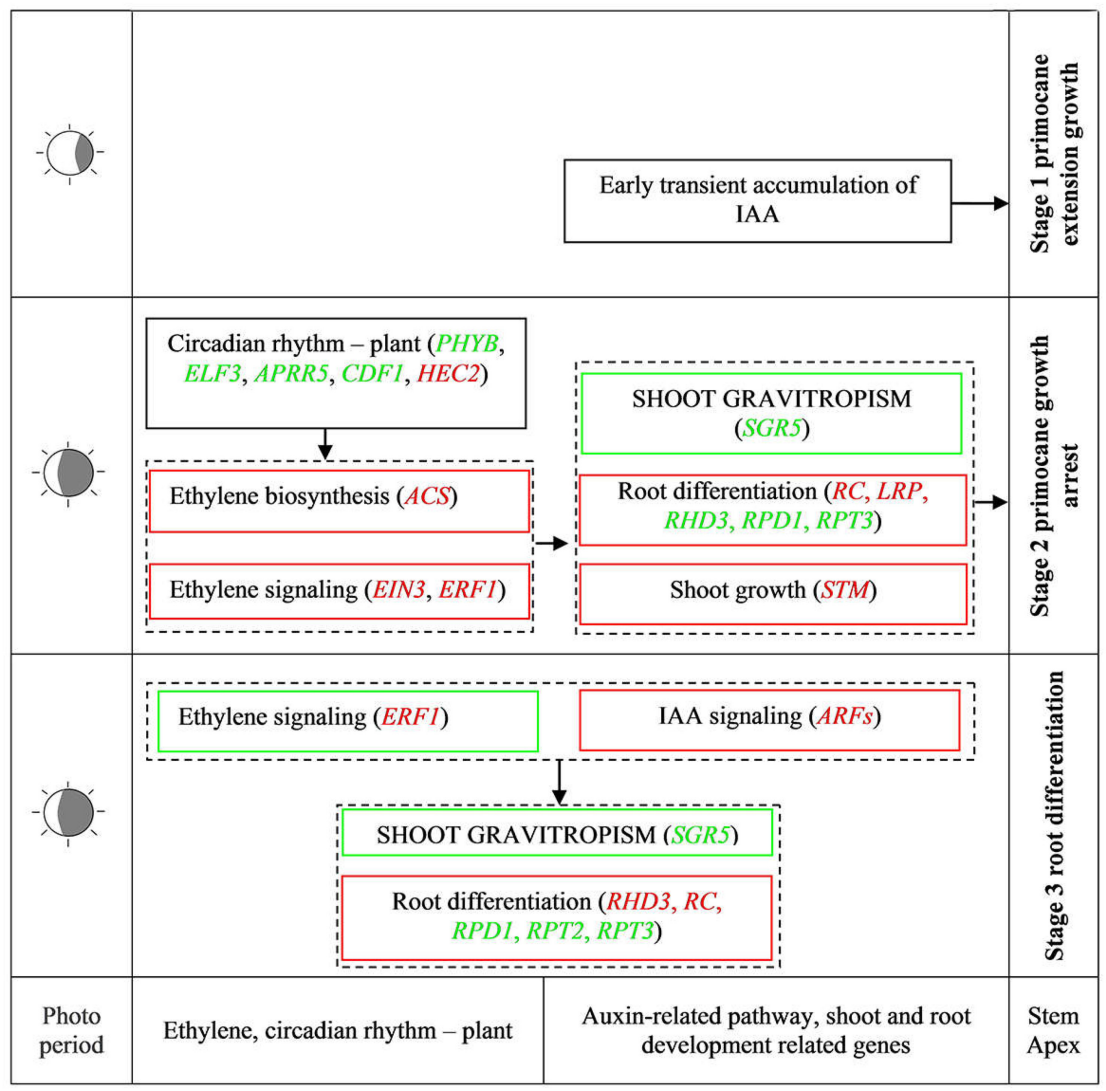
Simple model of important physiological units of spontaneous rooting of stem apex and regulative factors controlling ethylene, auxin, ABA, and circadian rhythm in AR formation. Green and red words in square frames represent down- and up-regulated DEGs, respectively. Green and red square frames represent down and up regulation pathways, respectively. ACS, 1-Aminocyclopropane-1-Carboxylate Synthase; AIR12, Auxin Induced in Roots 12; APRR5, Two-Component Response Regulator APRR5; ARFs, Auxin Response Factors; CDF1, Cycling Dof Factor 1; CTR1, Threonine-Protein Kinase CTR1; EIN3, Ethylene-Insensitive3; ELF3, Early Flowering 3; ERF1, Ethylene-Responsive Transcription Factor1; HEC2, HECATE 2; LRP, Lateral Root Primordium; PHYB, Phytochrome B; RC, ROOT CAP; RHD3, Root Hair Defective 3; RPD1, ROOT Primordium Defective 1; RPT, Root Phototropism; SGR5, Shoot Gravitropism 5; STM, Shoot Meristemless.

During AR formation at the primocane apex, a series of physiological changes occurred according to the developmental time point (Figure 2), including shoot growth arrest, geotropic growth of the shoot, root primordium differentiation at the apex (stage 2), root development, soil entry of roots, and daughter plant formation (stage 3). Our transcriptome comparisons revealed that a set of DEGs related to circadian rhythm—plant, plant hormone signal transduction, and shoot and root development acted coordinately to regulate this unique rooting process. Based on this evidence, we propose a simple model of AR formation at the primocane apex in which photoperiod acts as the environmental inducer (Figure 9). At stage 1, the primocane exhibits a normal extending growth pattern under a long photoperiod. The shorter photoperiod during stage 2 (approach of autumn) induces changes in the expression of genes related to circadian rhythm, including *PHYB, ELF3, APRR5, CDF1*, and *HEC2*. These DEGs inactivate auxin signaling via downregulation of *ARFs* and activate ethylene signaling via upregulation of *ACS, EIN2*, and *ERF1/2*. Inactive auxin and active ethylene cause growth arrest and changes in geotropism that may be necessary for subsequent root differentiation, which is induced by the expression of root development-related genes such as *RC, LRP, RPD1*, and *RHD3*. At stage 2 and 3, auxin signaling is active while ethylene signaling is inactive, and root differentiation continues and root formation begins under the control of *RHD3, RC, RPD1, RPT2*, and *RPT3*. In conclusion, as a plant with a unique rooting habit, not only typical rooting-related DEGs but also components of auxin and ethylene signaling pathways in addition to shoot growth- and circadian rhythm-related genes modulating shoot growth arrest and growth direction were found to be associated with the rooting process.

## Author contributions

JL and YC contributed to study conception and design, collection and/or assembly of data, data analysis and interpretation, and manuscript writing. YM, YZ, JX, and YS prepared samples, collected and/or assembled data, and analyzed and interpreted data.

### Conflict of interest statement

The authors declare that the research was conducted in the absence of any commercial or financial relationships that could be construed as a potential conflict of interest.
