# Supplementary material for: Comparative Transcriptome Analysis Reveal Candidate Genes Potentially Involved in Regulation of Primocane Apex Rooting in Raspberry (Rubus spp.)
Source: Front Plant Sci. 2017 Jun 13;8:1036. doi: 10.3389/fpls.2017.01036 (PMC5469044; doi:10.3389/fpls.2017.01036)
Supplement: Supplementary file 1 [file Table1.docx]

**Supplement Table 1** | Primers used for qRT-PCR assay

| Key target genes | Gene-specific Primers | | Amplicon size (bp) | Unigene ID |
| --- | --- | --- | --- | --- |
| *ACS* | F | 5’-ctgcactagctccaatggct-3’ | 102 | Unigene20575_All |
|  | R | 5’-gtgaccaaaacgcccttgac-3’ |  |  |
| *ARG7* | F | 5’-caggtgtcctcgtccgaatc-3’ | 89 | Unigene20551_All |
|  | R | 5’-agtcccctatctagccgtgg-3’ |  |  |
| *ERF1* | F | 5’ gtcgtaagctagggctgcat-3’ | 91 | Unigene13079_All |
|  | R | 5’- cgctgcggagataagggatt -3’ |  |  |
| [*SGR5*](http://www.baidu.com/link?url=sI2rC7gY2frlhnFeycxd1sJVr6J6jDB-Z1SNiriRQtl05tDfQbK0m228801vbNn5&wd=&eqid=9bc761740000ce740000000458733162) | F | 5’- atgccaaagccaagtgacct -3’ | 193 | Unigene12164_All |
|  | R | 5’- aatgcgttgcgtagtgttcg -3’ |  |  |
| [*RPT3*](http://www.baidu.com/link?url=u9htDRJ39XbbjsEw6qzNFlWl3KBUh-fwMX9Lat8sCObylvPd211u610mNv4dFbFn&wd=&eqid=b39eabbd0000cb360000000258733193) | F | 5’- ttctcatccacaaggcggtc -3’ | 111 | Unigene15061_All |
|  | R | 5’- actacgcctccaaatggctc -3’ |  |  |
| *RPD1* | F | 5’- cccactaccggttcattgct 3’ | 81 | Unigene20360_All |
|  | R | 5’- caaacgtgggatctctggct -3’ |  |  |
| *HISTONE H3* (*HTR3*) | F | 5’-ttccagagccatgcagttttg-3’ | 93 | AF304365.1 |
|  | R | 5’-tggcatgaatggcacagaga-3’ |  |  |
| *ACTIN* (*ACT*) | F | 5’-attgcagaccgtatgagcaaag3’ | 62 | GQ339772.1 |
|  | R | 5’-ggtgccacaaccttgatcttc-3’ |  |  |
